# Studies on a new K-symbol analytic functions generated by a modified K-symbol Riemann-Liouville fractional calculus

**DOI:** 10.1016/j.mex.2023.102398

**Published:** 2023-10-01

**Authors:** Ibtisam Aldawish, Rabha W. Ibrahim

**Affiliations:** a Department of Mathematics and Statistics, College of Science, IMSIU (Imam Mohammad Ibn Saud Islamic University), Riyadh, Saudi Arabia; b Mathematics Research Center, Department of Mathematics, Near East University, Near East Boulevard, PC: 99138, Nicosia /Mersin 10-Turkey, Turkey; c Department of Computer Science and Mathematics, Lebanese American University, Beirut, Lebanon; d Information and Communication Technology Research Group, Scientific Research Center, Al-Ayen University, Thi-Qar, Iraq

**Keywords:** K-symbol fractional calculus type Riemann-Liouville operators, Open unit disk, K-symbol fractional calculus, Fractional differential operator, Univalent function, Bounded turning function, Analytic function

## Abstract

Analytic functions are very helpful in many mathematical and scientific uses, such as complex integration, potential theory, and fluid dynamics, due to their geometric features. Particularly conformal mappings are widely used in physics and engineering because they make it possible to convert complex physical issues into simpler ones with simpler answers. We investigate a novel family of analytic functions in the open unit disk using the K-symbol fractional differential operator type Riemann-Liouville fractional calculus of a complex variable. For the analysis and solution of differential equations containing many fractional orders, it offers a potent mathematical framework. There are ongoing determinations to strengthen the mathematical underpinnings of K-symbol fractional calculus theory and investigate its applications in various fields.

• Normalization is presented for the K-symbol fractional differential operator. Geometric properties are offered of the proposed K-symbol fractional differential operator, such as the starlikeness property and hence univalency in the open unit disk.
• The formula of the Alexander integral involving the proposed operator is suggested and studied its geometric properties such as convexity.
• Examples are illustrated to fit our pure result. Here, the technique is based on the concepts of geometric function theory in the open unit disk, such as the subordination and Jack lemma.

## Graphical abstract

**Figure:**
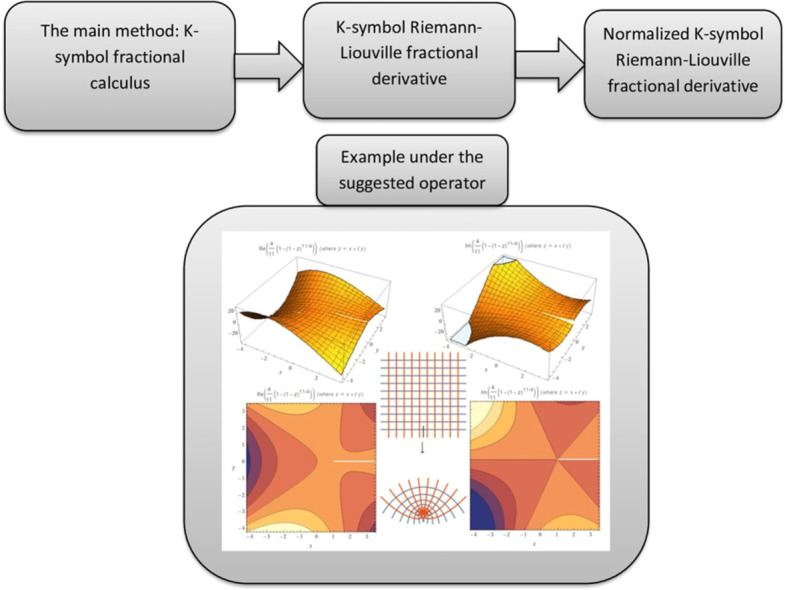

Specifications table

**Table:** 

Subject area:	Mathematics and Statistics
More specific subject area:	*Fractional Calculus*
Name of your method:	*K-symbol fractional calculus type Riemann-Liouville operators*
Name and reference of original method:	*[1] Diaz, R., and E. Pariguan. “On Hypergeometric Functions and Pochhammer K-symbol, Divulgaciones Matemticas, 15.” (2007): 179–192.*
Resource availability:	*N/A*

## Background

Multi fractional orders are included in the generalization of classical fractional calculus known as K-symbol fractional calculus [1]. The derivative and integral operators in conventional fractional calculus have been widened to non-integer orders, enabling the investigation of non-local and memory-dependent phenomena. The fractional orders directly are regarded as variables in K-symbol fractional calculus and can have many values at once. As a mathematical tool to represent complicated systems with effects of memory that are unable to be fully described by single fractional orders, the K-symbol fractional calculus framework was created. It offers a more adaptable and flexible method for modeling and evaluating these systems. A group of fractional orders, each linked to a value or factor, are represented by the K-symbol shorthand [2,3].

K-symbol fractional calculus facilitates the modeling of systems with various memory characteristics or heterogeneous qualities by taking into account numerous fractional orders. It enables the integration of numerous phenomenological or physical factors into a coherent framework. This can be especially helpful in fields like physics, engineering, and signal processing where memory-rich complicated systems are present [4], [5], [6]. Similar to classical fractional calculus, K-symbol fractional calculus includes fractional differentiation and integration as mathematical operations. However, the fractional orders are now denoted by the K-symbol and are not anymore fixed certainties. Algebraically, the K-symbol can be used to perform operations on the systems and functions that fractional differential equations explain. Investigation on the K-symbol fractional calculus is ongoing, and several theoretical advancements and applications are currently investigated. It offers a promising strategy for comprehending and simulating systems with intricate memory effects and has the ability to shed new light on a variety of phenomena’ behaviors [7], [8], [9].

Ultimately, the non-local and non-integer nature of fractional calculus creates geometric obstacles that make it necessary to develop new geometric interpretations and visualization methods in order to completely comprehend and make use of this mathematical framework in a variety of applications. To overcome these issues and deepen our understanding of fractional calculus' geometric foundations, investigators are still investigating and creating new strategies.

A subfield of complex analysis called geometric function theory investigates the geometric features and behavior of analytic functions in the complex plane. It covers subjects like conformal mappings, the Riemann mapping theorem, and the investigation of a number of geometric characteristics of analytic functions. By using this branch and its rich information, we proceed to discover more applications of the K-symbol fractional calculus of a complex variable in the open unit disk. In deduction, while working with structured data, analytical functions offer efficiency, simplicity, and readability. For processes including ranking, aggregate, time-series analysis, and comparative analysis, they are very helpful. By utilizing analytical functions, one may create SQL queries that are shorter, faster, and easier to manage for your data analysis occupations.

In this effort, we employ the normalized K-symbol Riemann-Liouville fractional operators of a complex variable to introduce a novel family of analytic functions in the open unit disk. We present a collection of geometric properties of the suggested operator by applying Jack lemma and the subordination concept. Note that Jack lemma is the most popular analyze method to discover the relation between the class of analytic functions and its properties. Moreover, the connection bounds (coefficient bounds) for the class are determined and conditions for the starlikeness and convexity are indicated including the Alexander operator. As a consequence and a comparison with previous works, some corollaries are recognized. All the figures are plotted by using Mathematica 13.3.

## Method details

1. K-symbol fractional calculus

**Fig. 1:**
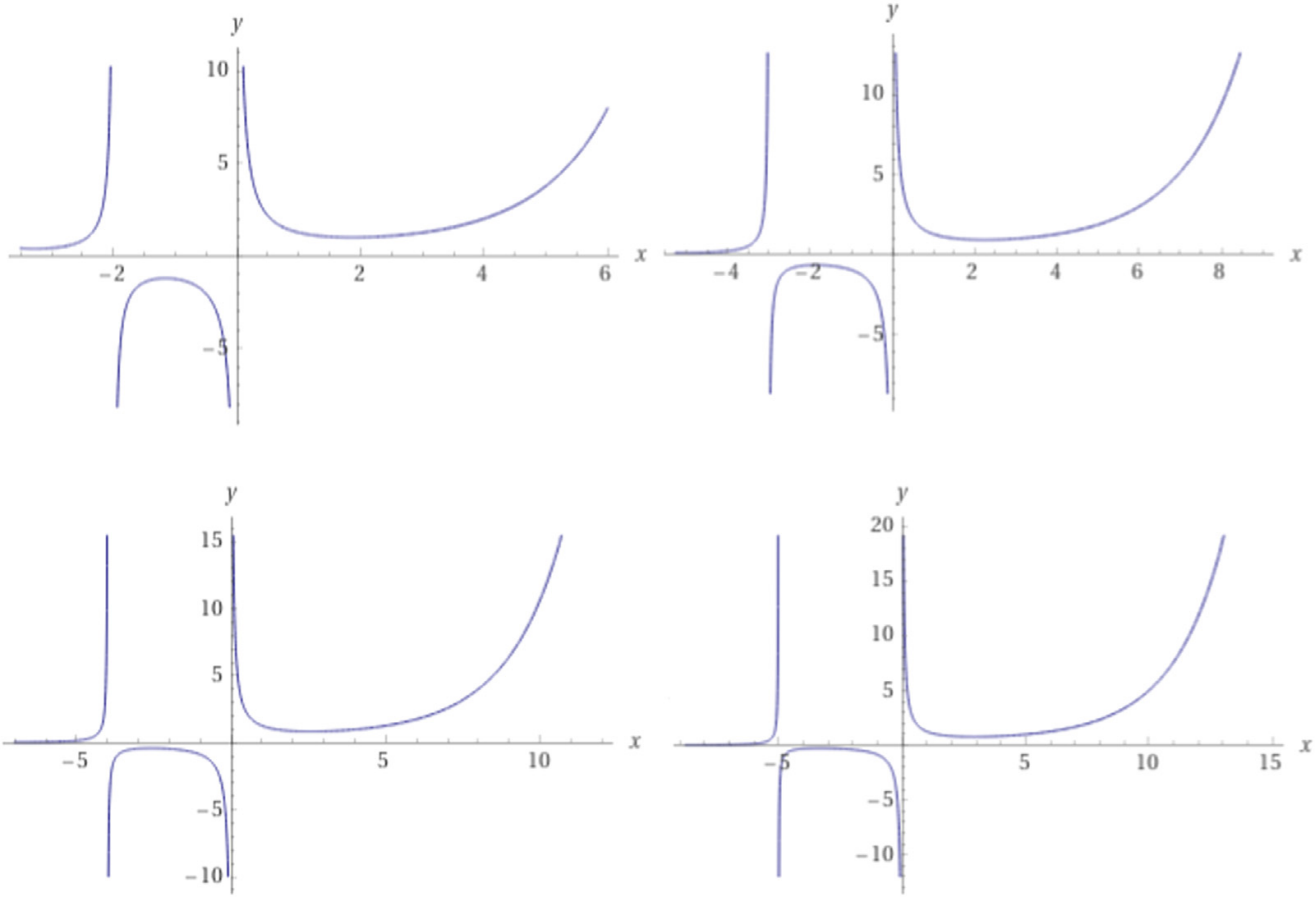
The plot of $\Gamma_{1}\left(u\right),\;\Gamma_{2}\left(u\right),\;\Gamma_{3}\left(u\right),\;\Gamma_{4}\left(u\right),\;$respectively.

The incentive's gamma function, also referred to as the K-symbol gamma function, is defined in the following manner [1] (see Fig. 1):

$$\Gamma_{k}\left(u\right)=\underset{m\to \infty }{\text{lim}}\frac{m!k^{m}{\left(mk\right)}^{\frac{u}{k}-1}}{{\left(u\right)}_{m,k}},\;\;k>0$$

where

$${\left(u\right)}_{m,k}≔u\left(u+k\right)\left(u+2k\right)...\left(u+\left(m-1\right)k\right)$$

and

$${\left(u\right)}_{m,k}=\frac{\Gamma_{k}\left(u+m\;k\right)}{\Gamma_{k}\left(u\right)}.$$

Moreover,

$$\Gamma \left(u\right)=\underset{k\to 1}{\text{lim}}\Gamma_{k}\left(u\right);\;\;\Gamma_{k}\left(u\right)=k^{u/k-1}\Gamma \left(u/k\right);\;\;\Gamma_{k}\left(u+k\right)=u\Gamma_{k}\left(u\right).$$

Definition 1 The design of the K-symbol Riemann-Liouville fractional integral is as follows:

$$L_{a,k}^{\gamma }\;g\left(y\right)=\frac{1}{k\Gamma_{k}\left(\gamma \right)}\int_{a}^{y}{\left(y-\upsilon \right)}^{\gamma /k-1}g\left(\upsilon \right)d\upsilon .$$

Moreover, for γ∈(0,1) the formula for the K-Riemann-Liouville singular kernel is

$$J_{k}^{\gamma }\left(\upsilon \right)=\frac{{\left(\upsilon \right)}^{\gamma /k-1}}{k\Gamma_{k}\left(\gamma \right)},\;a=0.$$

Note that, when g(y)=1, we have

$$L_{a,k}^{\gamma }\;g\left(y\right)=\frac{{\left(y-a\right)}^{\gamma /k}}{\Gamma_{k}\left(\gamma +k\right)},\;\gamma \in \left(0,1\right].$$

**Fig. 2:**
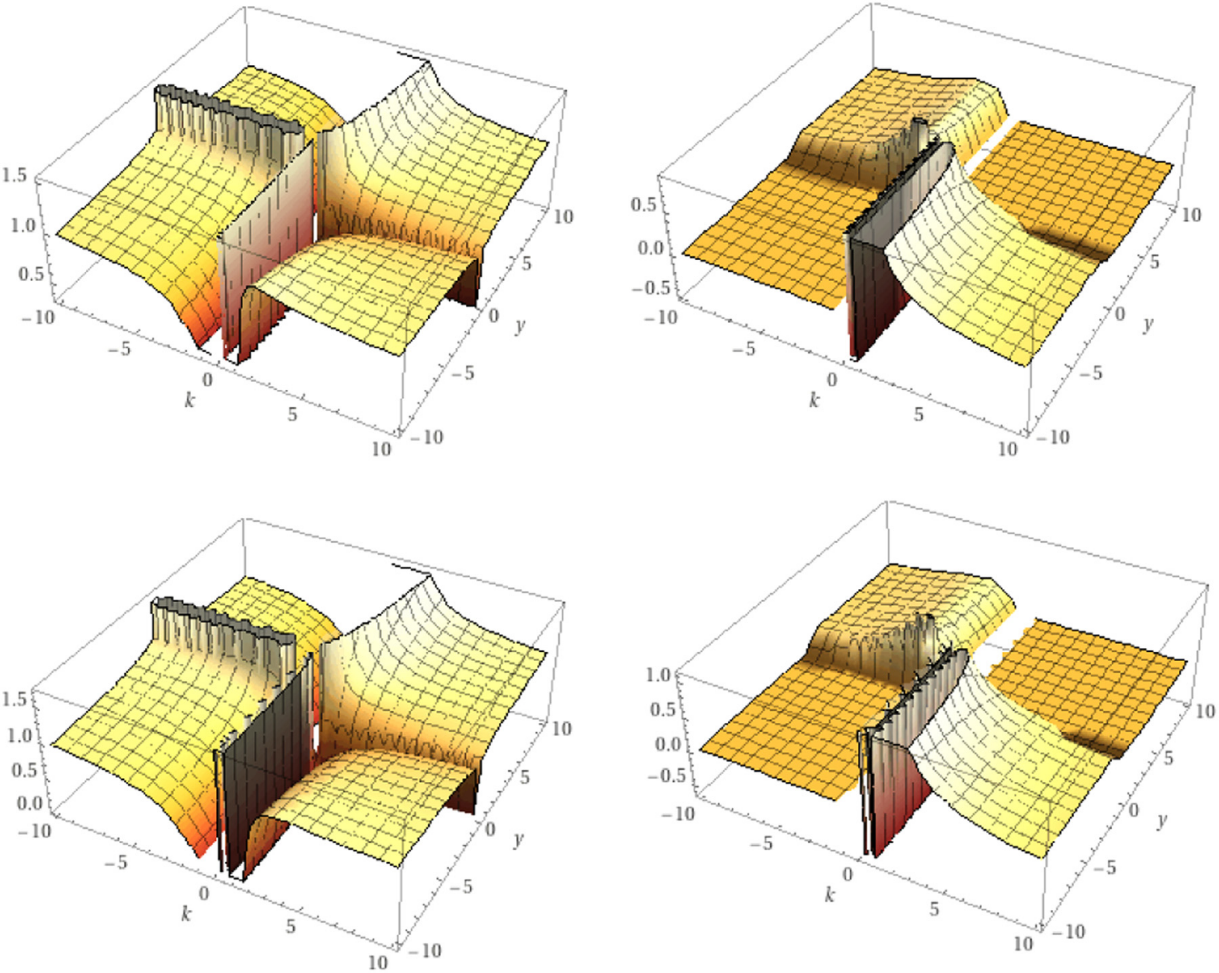
The real and the imaginary parts of the K-symbol integral $L_{k}^{0.5},L_{k}^{0.75}\;$respectively.

As an exception, when a=0, we obtain (see Fig. 2)

$$L_{k}^{\gamma }\;g\left(y\right)=\frac{1}{k\Gamma_{k}\left(\gamma \right)}\int_{0}^{y}{\left(y-\upsilon \right)}^{\gamma /k-1}g\left(\upsilon \right)d\upsilon .$$

Thus, when g(y)=1,a=0, we get

$$L_{k}^{\gamma }\;g\left(y\right)=\frac{y^{\gamma /k}}{\Gamma_{k}\left(\gamma +k\right)},\;\gamma \in \left(0,1\right]=\frac{y^{\frac{\gamma }{k}}}{k^{\frac{\gamma +k}{k}-1}\Gamma \left(\frac{\gamma +k}{k}\right)}.$$

Also, we have the following example, when $g\left(y\right)=y^{\frac{\alpha }{k}}$

$$\begin{array}{ccc} L_{k}^{\gamma }\;g\left(y\right) & = & \frac{\Gamma_{k}\left(\alpha +k\right)}{\Gamma_{k}\left(\alpha +\gamma +k\right)}y^{\frac{\alpha +\gamma }{k}},\;\alpha >0\\ & = & \frac{k^{\frac{\alpha +k}{k}-1}\Gamma \left(\frac{\alpha +k}{k}\right)}{k^{\frac{\alpha +\gamma +k}{k}-1}\Gamma \left(\frac{\alpha +\gamma +k}{k}\right)}y^{\frac{\alpha +\gamma }{k}}=k^{\frac{-\gamma }{k}}\left(\frac{\Gamma \left(\frac{\alpha +k}{k}\right)}{\Gamma \left(\frac{\alpha +\gamma +k}{k}\right)}\right)y^{\frac{\alpha +\gamma }{k}}. \end{array}$$

It is clear that when k=1, we receive the normal Riemann-Liouville fractional integral property

$$L_{1}^{\gamma }\;g\left(y\right)=\left(\frac{\Gamma \left(\alpha +1\right)}{\Gamma \left(\alpha +\gamma +1\right)}\right)y^{\alpha +\gamma }.$$

Definition 2 The K-symbol Riemann-Liouville fractional derivative is formulated by the following equality, when γ∈(0,1]:

$$\begin{array}{c} D_{k}^{\gamma }\;g\left(y\right)=\frac{d}{dy}\left(\;\;L_{k}^{1-\gamma }\;g\left(y\right)\right). \end{array}$$

Or, for more general case

$$\begin{array}{c} D_{k}^{\gamma }\;g\left(y\right)=\frac{d}{dy}\left(\;k\;L_{k}^{k-\gamma }\;g\left(y\right)\right). \end{array}$$

*Example 3.* the K-symbol derivative of g(y)=1 is

$$\begin{array}{ccc} D_{k}^{\gamma }\;g\left(y\right) & = & \frac{\left(1-\gamma \right)y^{\frac{-\left(k+\gamma -1\right)}{k}}}{k\Gamma_{k}\left(1-\gamma +k\right)}.\\ & = & \frac{\left(1-\gamma \right)y^{\frac{-\left(k+\gamma -1\right)}{k}}}{k^{\frac{1-\gamma +k}{k}}\Gamma \left(\frac{1-\gamma +k}{k}\right)}. \end{array}$$

**Fig. 3:**
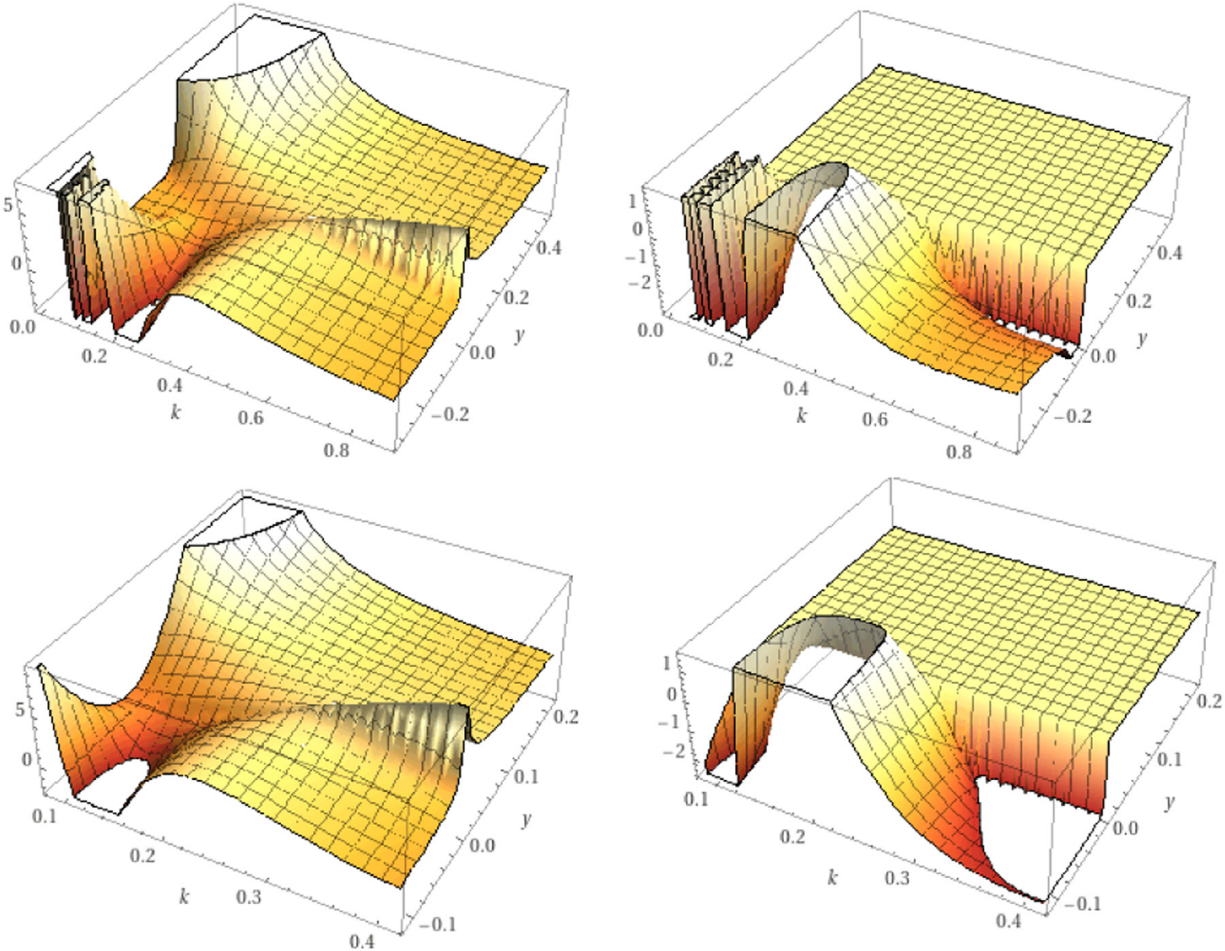
The real and the imaginary parts of the K-symbol integral $D_{k}^{0.5},D_{k}^{0.75}\;$respectively.

While, for $g\left(y\right)=y^{\alpha /k}$ we get (see Fig. 3)

$$D_{k}^{\gamma }\;g\left(y\right)=\left(\alpha +1-\gamma \right)k^{\frac{\gamma -1-k}{k}}\left(\frac{\Gamma \left(\frac{\alpha +k}{k}\right)}{\Gamma \left(\frac{\alpha -\gamma +k+1}{k}\right)}\right)y^{\frac{\alpha -\gamma }{k}}.$$

Clearly, when k=1, we obtain the normal fractional derivative

$$D_{1}^{\gamma }\;g\left(y\right)=\left(\frac{\Gamma \left(\alpha +1\right)}{\Gamma \left(\alpha -\gamma +1\right)}\right)y^{\alpha -\gamma },\;\gamma \in \left[0,1\right].$$

The K-symbol Caputo calculus is given by

(1) $${}^{C}D_{k}^{\gamma }g\left(y\right)=\left\{ \begin{array}{c} k^{w}L_{k}^{wk-\gamma }g^{\left(w\right)}\left(y\right)=\frac{k^{w-1}}{\Gamma_{k}\left(wk-\gamma \right)}\int_{0}^{y}\frac{g^{\left(w\right)}\left(\upsilon \right)}{{\left(y-\upsilon \right)}^{\gamma /k-w+1}}d\upsilon ,\;\left(w-1\right)k<\gamma <wk\\ \frac{d^{w}}{dy^{w}}g\left(y\right),\;wk=\gamma . \end{array}\right.$$

Keep in mind that we get the traditional Caputo derivative when k=1. Additionally, the Mittag-Leffler form function can be described in the following way dependent on the K-symbol gamma function:

$$\Xi_{\alpha ,\beta }^{k}\left(y\right)=\sum_{m=0}^{\infty }\frac{y^{m}}{\Gamma_{k}\left(\alpha m+\beta \right)}.$$

2. Geometric method

Assume that $\Lambda_{n}$ is the class of analytic functions ℘ in Δ≔{ζ∈C:|ζ|<1} with ${℘}^{\left(m\right)}\left(0\right)=0$ for m=0,1,...,n, where ${℘}^{\left(0\right)}\left(0\right)=℘\left(0\right).$ And that $\omega^{\left(0\right)}\left(\zeta \right)=\omega \left(\zeta \right),$ the set of n-dimensional Schwarz functions is defined, as follows:

$$S_{n}=\left\{\omega :\omega \;\;\text{is}\;\text{analytic},\;\left|\omega \left(\zeta \right)\right|<1\;\text{in}\;\Delta \;\text{and}\;\;\omega^{\left(m\right)}\left(0\right)=0\;\;\text{for}\;\;\;m=0,1,...,n\right\}.$$

Note that when n=0, we obtain the well-known the set of Schwarz functions. Analytic function ρ in Δ is in the normalized subclass Λ if it admits $\rho \left(0\right)=\rho {}^{\prime }\left(0\right)-1=0.$ This class contains all the univalent functions S⊂Λ. The class S involves two geometric functions, the convex functions in the set K, and the starlike functions in the set $S^{*}$. For η(ζ) and ϑ(ζ), if an analytic function ϖ(ζ)∈Δ occurs and satisfies the conditions ϖ(0)=0,|ϖ(ζ)|<1 and η(ζ)=ϑ(ϖ(ζ) then η(ζ) is stated to be subordinate to ϑ(ζ)(see[10]).

We request the following preliminary [11]:

Lemma 1 *(Jack Lemma) With*v(0)=0*, assume that*v(ζ)*is analytic in*Δ*. At the point*$\zeta_{0}\in \Delta$*, where*|v(ζ)|*reaches its maximum value on the circle*|ζ|=r,*the derivative satisfies the equality*$\zeta_{0}v\left(\zeta_{0}\right){}^{\prime }=℘\;v\left(\zeta_{0}\right)$*, where*℘≥1*is a real number*.

3. K-Symbol functional class of analytic functions

The collection of all functions that are analytic in a particular domain is represented by the symbolO, which stands for the functional class of analytic functions. The specific characteristics of this class are determined by the behavior of the function within the domain and by the characteristics of the domain itself. There are many different functions with smoothness and regularity qualities in the complex plane, and they are all included in the functional class of analytic functions. These functions are crucial to complicated analysis and have a wide range of uses in physics and mathematics. Functions with fractional derivatives of a specific order are within the fractional class of analytic functions. Fractional differential equations or fractional power series expansions can be used to model these functions. In this part, by using the K-symbol Riemann-Liouville fractional derivative $D_{k}^{\gamma }$ (similarly for ${}^{C}D_{k}^{\gamma }$).

Proposition 2 *For*g∈Λ*, where*Λ*indicates the class of normalized analytic functions in*Δ*taking the form*

$$g\left(\zeta \right)=\zeta +\sum_{n=2}^{\infty }g_{n}\zeta^{n},\;\zeta \in \Delta ,$$

then

$$D_{k}^{\gamma }g\left(\zeta \right)=\left(\frac{\Gamma \left(\frac{2k-\gamma +1}{k}\right)}{\left(k+1-\gamma \right)k^{\frac{\gamma -1-k}{k}}}\right)\zeta^{\frac{\gamma }{k}}D_{k}^{\gamma }g\left(\zeta \right)$$

is in Λ.

*Proof.* The K-symbol fractional derative is given, as follows:

$$\begin{array}{ccc} D_{k}^{\gamma }g\left(\zeta \right) & = & D_{k}^{\gamma }\left(\zeta +\sum_{n=2}^{\infty }g_{n}\zeta^{n}\right)=D_{k}^{\gamma }\zeta^{k/k}+\sum_{n=2}^{\infty }g_{n}D_{k}^{\gamma }\zeta^{nk/k}\\ & = & \left(k+1-\gamma \right)k^{\frac{\gamma -1-k}{k}}\left(\frac{\Gamma \left(\frac{2k}{k}\right)}{\Gamma \left(\frac{k-\gamma +k+1}{k}\right)}\right)\zeta^{\frac{k-\gamma }{k}}+\sum_{n=2}^{\infty }g_{n}\left(nk+1-\gamma \right)k^{\frac{\gamma -1-k}{k}}\left(\frac{\Gamma \left(\frac{nk+k}{k}\right)}{\Gamma \left(\frac{nk-\gamma +k+1}{k}\right)}\right)\zeta^{\frac{nk-\gamma }{k}}\\ & = & \left(k+1-\gamma \right)k^{\frac{\gamma -1-k}{k}}\left(\frac{\Gamma \left(2\right)}{\Gamma \left(\frac{2k-\gamma +1}{k}\right)}\right)\zeta^{\frac{k-\gamma }{k}}+\sum_{n=2}^{\infty }g_{n}\left(nk+1-\gamma \right)k^{\frac{\gamma -1-k}{k}}\left(\frac{\Gamma \left(n+1\right)}{\Gamma \left(\frac{\left(n+1\right)k-\gamma +1}{k}\right)}\right)\zeta^{\frac{nk-\gamma }{k}}\\ & = & \left(\frac{\left(k+1-\gamma \right)k^{\frac{\gamma -1-k}{k}}}{\Gamma \left(\frac{2k-\gamma +1}{k}\right)}\right)\zeta^{\frac{k-\gamma }{k}}+\sum_{n=2}^{\infty }g_{n}\left(nk+1-\gamma \right)k^{\frac{\gamma -1-k}{k}}\left(\frac{\Gamma \left(n+1\right)}{\Gamma \left(\frac{\left(n+1\right)k-\gamma +1}{k}\right)}\right)\zeta^{\frac{nk-\gamma }{k}}. \end{array}$$

To normalize the above function, we define the K-symbol normalized fractional derivative, as follows:

$$D_{k}^{\gamma }g\left(\zeta \right)≔\left(\frac{\Gamma \left(\frac{2k-\gamma +1}{k}\right)}{\left(k+1-\gamma \right)k^{\frac{\gamma -1-k}{k}}}\right)\zeta^{\frac{\gamma }{k}}D_{k}^{\gamma }g\left(\zeta \right).$$

Thus, we obtain the following K-symbol normalized fractional derivative:

(2) $$\begin{array}{ccc} D_{k}^{\gamma }g\left(\zeta \right) & ≔\zeta & +\sum_{n=2}^{\infty }g_{n}\left(\frac{\Gamma \left(\frac{2k-\gamma +1}{k}\right)}{\left(k+1-\gamma \right)k^{\frac{\gamma -1-k}{k}}}\right)\left(\left(nk+1-\gamma \right)k^{\frac{\gamma -1-k}{k}}\right)\left(\frac{\Gamma \left(n+1\right)}{\Gamma \left(\frac{\left(n+1\right)k-\gamma +1}{k}\right)}\right)\zeta^{n}\\ & ≔\zeta & +\sum_{n=2}^{\infty }\Theta_{n}\left(k\right)g_{n}\zeta^{n}, \end{array}$$

where

$$\Theta_{n}\left(k\right)≔\left(\frac{\Gamma \left(\frac{2k-\gamma +1}{k}\right)}{\left(k+1-\gamma \right)k^{\frac{\gamma -1-k}{k}}}\right)\left(\left(nk+1-\gamma \right)k^{\frac{\gamma -1-k}{k}}\right)\left(\frac{\Gamma \left(n+1\right)}{\Gamma \left(\frac{\left(n+1\right)k-\gamma +1}{k}\right)}\right).$$

Obviously, $D_{1}^{1}g\left(\zeta \right)$ presents the Salagean's differential operator [12], when $\Theta_{n}\left(1\right)=n$. Our aim is to study the geometric properties of the operator (2) with a connection with the main value k>0.

## Results

1. Auxiliary results

In this place, we investigate some geometric properties of (2) such as the starlikeness and convexity.

Proposition 3 *Let*ψ∈Λ.*If*

$$ℜ\left(1+\frac{\zeta \psi^{\prime \prime }\left(\zeta \right)}{\psi {}^{\prime }\left(\zeta \right)}\right)<\frac{b\left(3k+2\right)-k}{2\left(b+1\right)k},\;k\geq 1,b\in \left(1,2\right]$$

then ψ is starlike with

$$\frac{\zeta \psi {}^{\prime }\left(\zeta \right)}{\psi \left(\zeta \right)}≺\frac{1}{k}\left(\frac{b\left(k-\zeta \right)}{b-\zeta }\right),\;\zeta \in \Delta .$$

Moreover, the Alexander integral

$$\int_{0}^{\zeta }\left(\frac{\psi \left(y\right)}{y}\right)dy,\;\zeta \in \Delta$$

is convex.

Note that, in knot theory and three-dimensional topology, the Alexander integral operator and the corresponding Alexander polynomial are key tools. They assist mathematicians in studying and categorizing knots and connections, figuring out their characteristics, and examining their connections to other mathematical arrangements.

*Proof.* Let v be an analytic function in Δ such that

$$\left(\frac{\zeta \psi {}^{\prime }\left(\zeta \right)}{\psi \left(\zeta \right)}\right)=\frac{1}{k}\left(\frac{b\left(k-v\left(\zeta \right)\right)}{b-v\left(\zeta \right)}\right),\;\zeta \in \Delta .$$

Obviously, v is analytic in Δ with v(0)=0. It remains to show that |v(ζ)|<1.

The logarithmic derivative implies that

$$\left(1+\frac{\zeta \psi^{\prime \prime }\left(\zeta \right)}{\psi {}^{\prime }\left(\zeta \right)}\right)-\left(\frac{\zeta \psi {}^{\prime }\left(\zeta \right)}{\psi \left(\zeta \right)}\right)=-\frac{\zeta v{}^{\prime }\left(\zeta \right)}{k-v\left(\zeta \right)}+\frac{\zeta v{}^{\prime }\left(\zeta \right)}{b-v\left(\zeta \right)}.$$

This equivalent to

$$\left(1+\frac{\zeta \psi^{\prime \prime }\left(\zeta \right)}{\psi {}^{\prime }\left(\zeta \right)}\right)=-\frac{\zeta v{}^{\prime }\left(\zeta \right)}{k-v\left(\zeta \right)}+\frac{\zeta v{}^{\prime }\left(\zeta \right)}{b-v\left(\zeta \right)}+\frac{1}{k}\left(\frac{b\left(k-v\left(\zeta \right)\right)}{b-v\left(\zeta \right)}\right),\;\zeta \in \Delta .$$

Hence, we have the following real part inequality

$$ℜ\left(1+\frac{\zeta \psi^{\prime \prime }\left(\zeta \right)}{\psi {}^{\prime }\left(\zeta \right)}\right)=ℜ\left(-\frac{\zeta v{}^{\prime }\left(\zeta \right)}{k-v\left(\zeta \right)}+\frac{\zeta v{}^{\prime }\left(\zeta \right)}{b-v\left(\zeta \right)}+\frac{1}{k}\left(\frac{b\left(k-v\left(\zeta \right)\right)}{b-v\left(\zeta \right)}\right)\right)<\frac{b\left(3k+2\right)-k}{2\left(b+1\right)k}.$$

Now, by Lemma 1, there occurs a point $\zeta_{0}$ such that$\;\zeta_{0}v{}^{\prime }\left(\zeta_{0}\right)=℘v\left(\zeta_{0}\right)$, ℘≥1 $v\left(\zeta_{0}\right)=e^{i\theta }$ and

$$\underset{\left|\zeta \right|\leq \left|\zeta_{0}\right|}{\text{max}}\left|v\left(\zeta \right)\right|=\left|v\left(\zeta_{0}\right)\right|=1.$$

As a consequence, we get

$$\begin{array}{ccc} & & ℜ\left(1+\frac{\zeta_{0}\psi^{\prime \prime }\left(\zeta_{0}\right)}{\psi {}^{\prime }\left(\zeta_{0}\right)}\right)\\ & & =ℜ\left(-\frac{\zeta_{0}v{}^{\prime }\left(\zeta_{0}\right)}{k-v\left(\zeta_{0}\right)}+\frac{\zeta_{0}v{}^{\prime }\left(\zeta_{0}\right)}{b-v\left(\zeta_{0}\right)}+\frac{1}{k}\left(\frac{b\left(k-v\left(\zeta_{0}\right)\right)}{b-v\left(\zeta_{0}\right)}\right)\right)\\ & & =ℜ\left(-\frac{℘e^{i\theta }}{k-e^{i\theta }}+\frac{℘e^{i\theta }}{b-e^{i\theta }}+\frac{1}{k}\left(\frac{b\left(k-e^{i\theta }\right)}{b-e^{i\theta }}\right)\right)\\ & & =ℜ\left(\frac{b\left(b-\left(℘+1\right)k\right)}{k\left(-b+e^{i\theta }\right)}+\frac{℘}{\left(-1+e^{i\theta }\right)}+\frac{b}{k}\right)\\ & & =-\frac{℘cos^{2}\left(\theta \right)}{{\left(b-cos\left(\theta \right)\right)}^{2}+sin^{2}\left(\theta \right)}+\frac{℘bcos\left(\theta \right)}{{\left(b-cos\left(\theta \right)\right)}^{2}+sin^{2}\left(\theta \right)}-\frac{℘sin^{2}\left(\theta \right)}{{\left(b-cos\left(\theta \right)\right)}^{2}+sin^{2}\left(\theta \right)}\\ & & +\frac{℘cos^{2}\left(\theta \right)}{sin^{2}\left(\theta \right)+{\left(1-cos\left(\theta \right)\right)}^{2}}+\frac{℘sin^{2}\left(\theta \right)}{sin^{2}\left(\theta \right)+{\left(1-cos\left(\theta \right)\right)}^{2}}-\frac{℘cos\left(\theta \right)}{sin^{2}\left(\theta \right)+{\left(1-cos\left(\theta \right)\right)}^{2}}\\ & & +\frac{b^{2}}{{\left(b-cos\left(\theta \right)\right)}^{2}+sin^{2}\left(\theta \right)}-\frac{b^{2}cos\left(\theta \right)}{k\left({\left(b-cos\left(\theta \right)\right)}^{2}+sin^{2}\left(\theta \right)\right)}-\frac{bcos\left(\theta \right)}{{\left(b-cos\left(\theta \right)\right)}^{2}+sin^{2}\left(\theta \right)}\\ & & +\frac{b\;cos^{2}\left(\theta \right)}{\left(k\left({\left(b-cos\left(\theta \right)\right)}^{2}+sin^{2}\left(\theta \right)\right)\right)}+\frac{b\;sin^{2}\left(\theta \right)}{k\left({\left(b-cos\left(\theta \right)\right)}^{2}+sin^{2}\left(\theta \right)\right)}\\ & & \geq -\frac{℘cos^{2}\left(\theta \right)}{{\left(b-cos\left(\theta \right)\right)}^{2}+sin^{2}\left(\theta \right)}+\frac{℘bcos\left(\theta \right)}{{\left(b-cos\left(\theta \right)\right)}^{2}+sin^{2}\left(\theta \right)}-\frac{℘sin^{2}\left(\theta \right)}{{\left(b-cos\left(\theta \right)\right)}^{2}+sin^{2}\left(\theta \right)}\\ & & +\frac{℘cos^{2}\left(\theta \right)}{sin^{2}\left(\theta \right)+{\left(1-cos\left(\theta \right)\right)}^{2}}+\frac{℘sin^{2}\left(\theta \right)}{sin^{2}\left(\theta \right)+{\left(1-cos\left(\theta \right)\right)}^{2}}-\frac{℘cos\left(\theta \right)}{sin^{2}\left(\theta \right)+{\left(1-cos\left(\theta \right)\right)}^{2}}\\ & & +\frac{b^{2}}{{\left(b-cos\left(\theta \right)\right)}^{2}+sin^{2}\left(\theta \right)}-\frac{b^{2}cos\left(\theta \right)}{\left({\left(b-cos\left(\theta \right)\right)}^{2}+sin^{2}\left(\theta \right)\right)}-\frac{bcos\left(\theta \right)}{{\left(b-cos\left(\theta \right)\right)}^{2}+sin^{2}\left(\theta \right)}\\ & & +\frac{b\;cos^{2}\left(\theta \right)}{\left(k\left({\left(b-cos\left(\theta \right)\right)}^{2}+sin^{2}\left(\theta \right)\right)\right)}+\frac{b\;sin^{2}\left(\theta \right)}{k\left({\left(b-cos\left(\theta \right)\right)}^{2}+sin^{2}\left(\theta \right)\right)} \end{array}$$

The right side of the above inequality can be approximated by the series when ℘≥1

$$\begin{array}{ccc} ℜ\left(1+\frac{\zeta_{0}\psi^{\prime \prime }\left(\zeta_{0}\right)}{\psi {}^{\prime }\left(\zeta_{0}\right)}\right) & \geq & \frac{℘\left(b^{2}-1\right)}{2b^{2}-4bcos\left(\theta \right)+2}+\frac{b\left(-\left(b+k\right)cos\left(\theta \right)+bk+1\right)}{k\left(b^{2}-2bcos\left(\theta \right)+1\right)}\\ & \geq & \frac{\left(b^{2}-1\right)}{2b^{2}-4bcos\left(\theta \right)+2}+\frac{b\left(-\left(b+k\right)cos\left(\theta \right)+bk+1\right)}{k\left(b^{2}-2bcos\left(\theta \right)+1\right)}. \end{array}$$

Now by considering cos(θ)=−1, we have

$$\begin{array}{cc} ℜ\left(1+\frac{\zeta_{0}\psi^{\prime \prime }\left(\zeta_{0}\right)}{\psi {}^{\prime }\left(\zeta_{0}\right)}\right) & \geq \frac{b\left(3k+2\right)-k}{2\left(b+1\right)k} \end{array}$$

and this is a contradiction. Thus we obtain |v(ζ)|<1 for all ζ∈Δ. This implies

$$\frac{\zeta \psi {}^{\prime }\left(\zeta \right)}{\psi \left(\zeta \right)}≺\frac{1}{k}\left(\frac{b\left(k-\zeta \right)}{b-\zeta }\right),\;\;\zeta \in \Delta .$$

As a consequence ψ is starlike if and only if the integral

$$\int_{0}^{\zeta }\left(\frac{\psi \left(y\right)}{y}\right)dy,\;\;\zeta \in \Delta$$

is convex. ◻

The next outcome can be located in [13], when k=1.

Corollary 4 *Let*ψ∈Λ.*If*

$$ℜ\left(1+\frac{\zeta \psi^{\prime \prime }\left(\zeta \right)}{\psi {}^{\prime }\left(\zeta \right)}\right)<\frac{5b-1}{2\left(b+1\right)},b\in \left(1,2\right]$$

then ψ is starlike with

$$\frac{\zeta \psi {}^{\prime }\left(\zeta \right)}{\psi \left(\zeta \right)}≺\left(\frac{b\left(1-\zeta \right)}{b-\zeta }\right),\;\;\zeta \in \Delta .$$

Moreover, the integral

$$\int_{0}^{\zeta }\left(\frac{\psi \left(y\right)}{y}\right)dy,\;\;\zeta \in \Delta$$

is convex.

The next outcome can be located in [14], when k=1 and b=2.

Corollary 5 *Let*ψ∈Λ.*If*

$$ℜ\left(1+\frac{\zeta \psi^{\prime \prime }\left(\zeta \right)}{\psi {}^{\prime }\left(\zeta \right)}\right)<\frac{3}{2},\;\;\zeta \in \Delta$$

then ψ is starlike with

$$\frac{\zeta \psi {}^{\prime }\left(\zeta \right)}{\psi \left(\zeta \right)}≺\left(\frac{2\left(1-\zeta \right)}{2-\zeta }\right),\;\;\zeta \in \Delta .$$

Moreover, the Alexander integral

$$\int_{0}^{\zeta }\left(\frac{\psi \left(y\right)}{y}\right)dy,\;\zeta \in \Delta$$

is convex.

Corollary 6 *Let the assumptions of* Proposition 3 *hold. Then*

$$\begin{array}{c} \left|\frac{\zeta \psi {}^{\prime }\left(\zeta \right)}{\psi \left(\zeta \right)}-\frac{bk}{k+b}\right|<\frac{bk}{k+b}. \end{array}$$

*Proof.* Putting

$$\left(\frac{\zeta \psi {}^{\prime }\left(\zeta \right)}{\psi \left(\zeta \right)}\right)=\frac{1}{k}\left(\frac{b\left(k-v\left(\zeta \right)\right)}{b-v\left(\zeta \right)}\right),\;\;\zeta \in \Delta ,$$

where v be an analytic function in Δ. A computation yields

$$\begin{array}{ccc} v\left(\chi \right) & = & \frac{bk\left(1-\frac{\zeta \psi {}^{\prime }\left(\zeta \right)}{\psi \left(\zeta \right)}\right)}{b-k\frac{\zeta \psi {}^{\prime }\left(\zeta \right)}{\psi \left(\zeta \right)}}\\ & = & \frac{bk\left(\frac{\zeta \psi {}^{\prime }\left(\zeta \right)}{\psi \left(\zeta \right)}-1\right)}{k\frac{\zeta \psi {}^{\prime }\left(\zeta \right)}{\psi \left(\zeta \right)}-b}, \end{array}$$

where |v|<1. We conclude the result. ◻

When k=1, we obtain the result in [13]. Moreover, when k=1 and b=2, we get a result in [14].

Example 7 Define the following analytic function:

$$\begin{array}{ccc} & & \psi \left(\zeta \right)\\ & & =\left(\frac{b+k}{3bk-1}\right)\left(1-{\left(1-\zeta \right)}^{\frac{3bk-1}{b+k}}\right)\\ & & =\zeta +\frac{\left(\zeta^{2}\left(-3bk+b+k+1\right)\right)}{\left(2\left(b+k\right)\right)}+\frac{\left(\zeta^{3}\left(b\left(3k-2\right)-2k-1\right)\left(b\left(3k-1\right)-k-1\right)\right)}{\left(6{\left(b+k\right)}^{2}\right)}\\ & & -\frac{\left(\zeta^{4}\left(\left(3b\left(k-1\right)-3k-1\right)\left(b\left(3k-2\right)-2k-1\right)\left(b\left(3k-1\right)-k-1\right)\right)\right)}{\left(24{\left(b+k\right)}^{3}\right)}\\ & & +\frac{\left(\zeta^{5}\left(3b\left(k-1\right)-3k-1\right)\left(b\left(3k-4\right)-4k-1\right)\left(b\left(3k-2\right)-2k-1\right)\left(b\left(3k-1\right)-k-1\right)\right)}{\left(120{\left(b+k\right)}^{4}\right)}\\ & & -\frac{\zeta^{6}\left(\left(3b\left(k-1\right)-3k-1\right)\left(b\left(3k-5\right)-5k-1\right)\left(b\left(3k-4\right)-4k-1\right)\left(b\left(3k-2\right)-2k-1\right)\left(b\left(3k-1\right)-k-1\right)\right)}{720{\left(b+k\right)}^{5}}\\ & & +O\left(\zeta^{7}\right). \end{array}$$

It is clear that ψ is a normalized analytic function in Δ. A calculation implies that

$$\frac{\zeta \psi {}^{\prime }\left(\zeta \right)}{\psi \left(\zeta \right)}=\frac{\zeta \left(3bk-1\right){\left(1-\zeta \right)}^{\frac{3bk-1}{\left(b+k\right)}-1}}{\left(b+k\right)\left(1-{\left(1-\zeta \right)}^{\frac{3bk-1}{b+k}}\right)}$$

and

$$1+\frac{\zeta \psi^{\prime \prime }\left(\zeta \right)}{\psi {}^{\prime }\left(\zeta \right)}=1-\frac{\frac{3bk-1}{b+k}-1}{1-\zeta }.$$

**Fig. 4:**
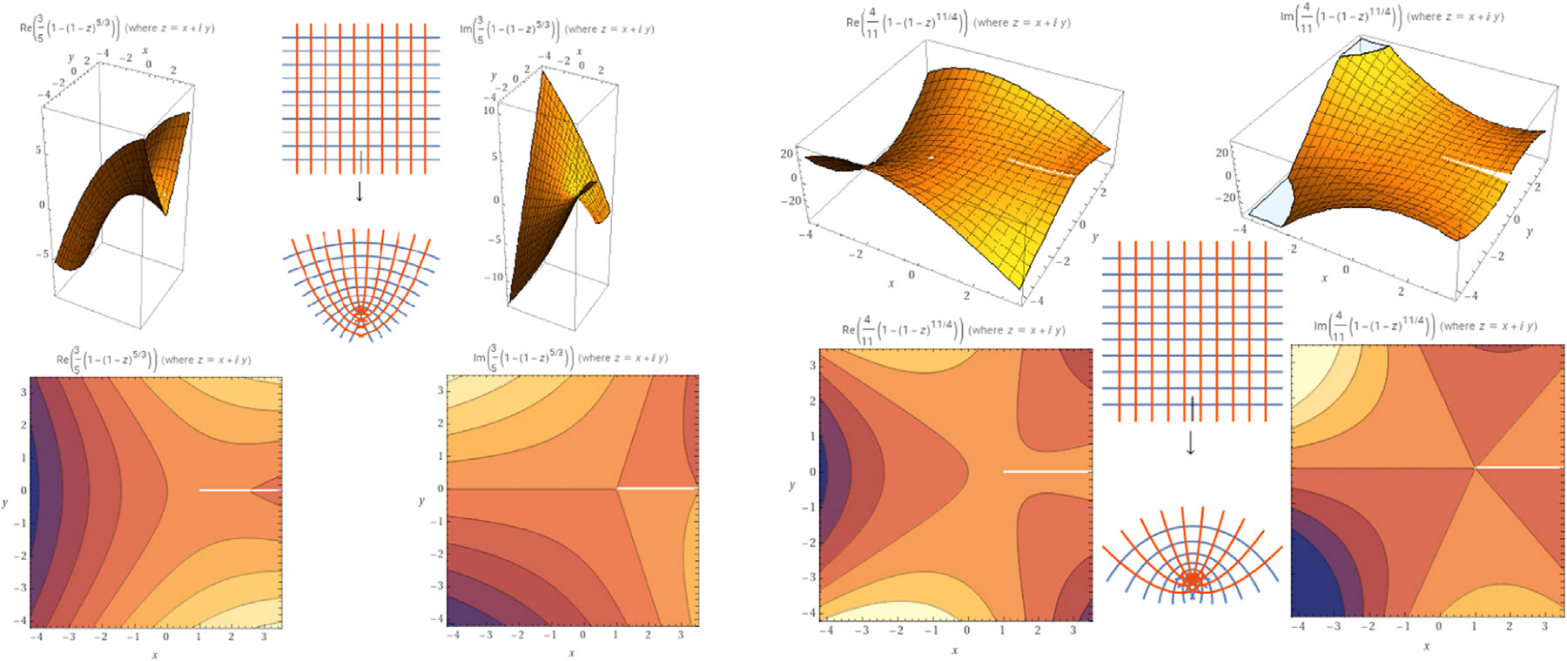
The plot of ψ when *b* = 2, *k* = 1 and *b* = 2, *k* = 2 respectively.

Thus, we have the following inequality

$$\begin{array}{ccc} ℜ\left(1+\frac{\zeta \psi^{\prime \prime }\left(\zeta \right)}{\psi {}^{\prime }\left(\zeta \right)}\right) & = & ℜ\left(1-\frac{\frac{3bk-1}{b+k}-1}{1-\zeta }\right)\\ & = & \frac{1}{2}\left(1-\frac{3bk-1}{b+k}\right)+1,\;\;\zeta \to -1\\ & < & \frac{b\left(3k+2\right)-k}{2\left(b+1\right)k} \end{array}$$

whenever b∈(1,2] and k>0. Hence, the function is starlike (see Fig. 4). Moreover, a calculation gives

$$\begin{array}{ccc} & & \int_{0}^{\zeta }\left(\frac{\psi \left(y\right)}{y}\right)dy\\ & & =\int_{0}^{\zeta }\frac{\left(b+k\right)(1-{\left(1-y\right)}^{\frac{3bk-1}{b+k}}}{\left(3bk-1\right)y}dy\\ & & =\frac{\left(b+k\right)\left(\frac{\left(b+k\right){\left(1-\zeta \right)}^{\frac{3bk+b+k-1}{b+k}}{\;}_{2}F_{1}\left(1,\frac{\left(3kb+b+k-1\right)}{\left(b+k\right)},\frac{\left(2k+b\left(3k+2\right)-1\right)}{\left(b+k\right)},1-\zeta \right)}{\left(3bk+b+k-1\right)}+\text{log}\left(\zeta \right)\right)}{\left(3bk-1\right)} \end{array}$$

which is convex in Δ. Note that ${}_{2}F_{1}\left(.\right)$ indicates the hypergeometric function.

Definition 3 A binary operation that can be applied to matrices or vectors, the Hadamard product, additionally referred to as the element-wise product, entails multiplying each element of one with its corresponding element in the other. The Hadamard product is also characterized via the formula of analytical processes [15]:

$$f_{1}\left(\zeta \right)*f_{2}\left(\zeta \right)=\left(\sum_{n=0}^{\infty }p_{n}\zeta^{n}\right)*\left(\sum_{n=0}^{\infty }q_{n}\zeta^{n}\right)=\sum_{n=0}^{\infty }p_{n}q_{n}\zeta^{n},\;\;\zeta \in \Delta .$$

In view of Hadamard product the fractional derivative $\left[D_{k}^{\gamma }g\left(\zeta \right)\right]$ can be viewed as a convolution operator, as follows:

$$\left[D_{k}^{\gamma }g\left(\zeta \right)\right]=\left[D_{k}^{\gamma }*g\right]\left(\zeta \right).$$

Remark 8 Note that

$$\left[D_{k}^{\gamma }g\left(\zeta \right)\right]{}^{\prime }=\left[D_{k}^{\gamma }\;*\zeta g^{\prime }\right]\left(\zeta \right),\;{\left[D_{k}^{\gamma }g\left(\zeta \right)\right]}^{\prime \prime }=\left[\zeta \left(D_{k}^{\gamma }\right){}^{\prime }*\zeta g^{\prime }\right]\left(\zeta \right).$$

Theorem 9 *Let*g∈Λ.*If*b∈(1,2]*,*k≥1*and*

$$ℜ\left(1+\frac{\zeta \left[\zeta \left(D_{k}^{\gamma }\right){}^{\prime }*\zeta g^{\prime }\right]\left(\zeta \right)}{\left[D_{k}^{\gamma }\left(\zeta \right)*\zeta g{}^{\prime }\left(\zeta \right)\right]}\right)<\frac{b\left(3k+2\right)-k}{2\left(b+1\right)k},\;\;k\geq 1,b\in \left(1,2\right]$$

then $\psi =[D_{k}^{\gamma }g]$ is starlike with

$$\frac{\zeta \left[D_{k}^{\gamma }\left(\zeta \right)*\zeta g{}^{\prime }\left(\zeta \right)\right]}{\left[D_{k}^{\gamma }g\left(\zeta \right)\right]}≺\frac{1}{k}\left(\frac{b\left(k-\zeta \right)}{b-\zeta }\right),\;\zeta \in \Delta .$$

Moreover, the Alexander integral

$$\int_{0}^{\zeta }\left(\frac{\left[D_{k}^{\gamma }g\right]\left(y\right)}{y}\right)dy,\;\zeta \in \Delta$$

is convex.

*Proof.* By using Proposition 2, Remark 8 and Proposition 3, we obtain the outcomes. ◻

## Conclusion

**Fig. 5:**
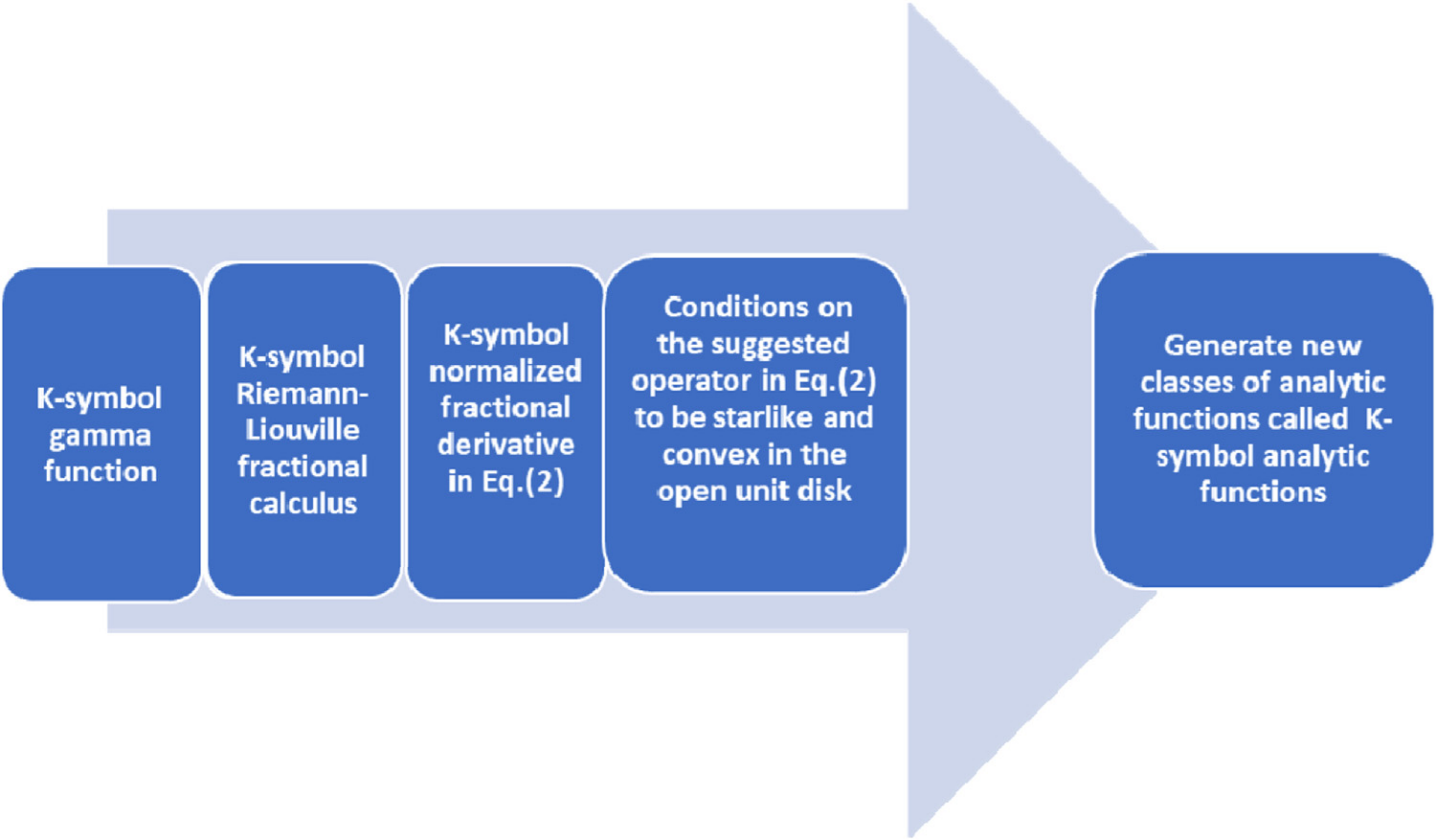
The process of the mathematical model that is studied geometrically.

To describe systems with memory and complex dynamics, K-symbol fractional calculus has been used in a number of disciplines, including physics, engineering, and signal processing. For the analysis and solution of differential equations containing many fractional orders, it offers a potent mathematical framework. There are also ongoing efforts to strengthen the mathematical underpinnings of K-symbol fractional calculus theory and investigate its applications in various fields (see Fig. 5). From above, we showed the efficiency of this calculus in terms of a normalized class of analytic functions. Based on this concept, we created a new generation of analytic functions in the open unit disk and studied its geometric properties. A K-symbol class of analytic functions is formulated by using the K-symbol fractional differential operator type Riemann-Liouville derivative. Moreover, some geometric properties of Alexander integral are presented.

## Ethics statements

The platforms’ data redistribution policies were complied with.

## Funding statement

This work was supported and funded by the Deanship of Scientific Research at Imam Mohammad Ibn Saud Islamic University (IMSIU) (grant number IMSIU-RP23001).

## CRediT authorship contribution statement

**Ibtisam Aldawish:** Conceptualization, Methodology, Writing – original draft. **Rabha W. Ibrahim:** Visualization, Investigation, Software, Writing – original draft.

## Declaration of Competing Interest

The authors declare that they have no known competing financial interests or personal relationships that could have appeared to influence the work reported in this paper.

## Data Availability

No data was used for the research described in the article.
